# Barriers and facilitators to ethical co-production with Autistic people with an eating disorder

**DOI:** 10.1186/s40337-024-01076-y

**Published:** 2024-08-09

**Authors:** Emy Nimbley, Ellen Maloney, Kyle Buchan, Michelle Sader, Karri Gillespie-Smith, Fiona Duffy

**Affiliations:** 1 Department of Clinical Psychology, School of Health in Social Science, University of Edinburgh, Edinburgh, EH8 9AG UK; 2 Eating Disorders and Autism Collaborative (EDAC), University of Edinburgh, Edinburgh, UK; 3 School of Medicine, Medical Sciences and Nutrition, University of Aberdeen, Aberdeen, UK

## Abstract

**Background:**

Co-production is the collaboration between researchers and the lived experience community in designing, conducting and sharing research. The importance of co-production is increasingly advocated in both the autism and eating disorder fields. Despite this, there remains a lack of clarity at how to define, apply and conduct ethical co-production. Understanding common challenges and what we can do to overcome these challenges are integral to ensuring ethical and meaningful research with Autistic people with an eating disorder. The current study therefore explored: What are the barriers and facilitators to ethical co-production with Autistic people with an ED?

**Methods:**

Five workshops were conducted with 30 collaborators exploring barriers and facilitators to ethical co-production. Synchronous (online workshops) and asynchronous (offline discussion forum) data was analysed using thematic analysis. Themes were co-produced by a neurotypical and Autistic researcher with lived/living experience of an eating disorder.

**Results:**

Four themes were identified that explored barriers to ethical co-production: unequal partnerships, the inaccessibility of research, excluded by diagnoses and communication differences. Three themes were identified with regards to facilitators of ethical co-production: shared power (with sub-themes relationships, not roles and creative compensation), clarity and transparency and autism-affirming approaches.

**Discussion:**

Conducting ethical co-production with Autistic people with eating disorders has the potential to generate meaningful research that can be translated into improving the lives of the Autistic and eating disorder community. To achieve this, co-production teams should strive towards shared power and long-term relationships, adapting for communication differences and preferences and operating firmly within an autism-affirming framework. It is hoped that study findings will inspire collaboration, discussion and novel, translatable research.

## Abstract (plain language summary)

Co-production is the collaboration between researchers and the lived experience community in designing, conducting and sharing research. This study brought together Autistic people with lived/living experience of an eating disorder (ED), researchers, clinicians, third sector organisations and parents/carers to understand what the barriers (challenges) and facilitators (how to overcome these challenges) were to ethical co-production with Autistic people with an eating disorder. Common barriers were found to be unequal partnerships, difficulty accessing research, feeling or being excluded by a reliance on diagnoses and the impact of communication differences. Facilitators were felt to be a shared power dynamic, focusing on establishing relationships not just tokenistic roles and creating fair and person-centred compensation. Facilitators were also adapting for communication differences and moving away from harmful medicalised and ableists models, towards autism-affirming practice. It is hoped that the study will encourage discussion and positive co-production relationships between autism and ED researchers and the Autistic and ED community. It is also hoped that that this approach will lead to more meaningful research that will ultimately improve the lives of Autistic people with an ED.

## Introduction

Autistic individuals typically show differences in social communication, repetitive patterns of behaviour and sensory processing compared to their neurotypical peers 3. Autistic individuals also experience a broad range of mental health difficulties [53], including eating disorders (EDs). It is estimated that around a third of those with anorexia nervosa (AN) demonstrate elevated Autistic traits [58, 65], and these elevated traits have also been reported across the range of feeding and eating disorders [50], such as bulimia nervosa (BN), binge eating disorder (BED) and avoidant restrictive food intake disorder (ARFID) [16, 21, 29]. While notably less research has explored prevalence estimates of disordered eating in Autistic samples, higher rates of EDs in Autistic people have been reported when compared to their neurotypical peers [22, 55].

Beyond establishing prevalence estimates however, research into underlying causes and mechanisms remain inconclusive. A wide range of social [6, 24, 36, 56, 57], emotional [25, 37, 63], sensory [1, 28, 45] and cognitive [30, 47] factors have been implicated (see [1] for a recent review). Several attempts have been made to propose ED models drawing on autism-specific mechanisms [8, 11, 26], however understandings of this overlap remain elusive. The implications of this are keenly highlighted by studies that have explored the impact of EDs on Autistic individuals. It has been reported that Autistic people with an ED report increased risk of admission to inpatient services [43, 46, 66], poorer experiences of ED services [4, 27], as well as poorer psychosocial outcomes such as significantly elevated rates of anxiety and reduced occupational functioning [33, 43]. Thus, there is a current lack of research-driven understanding of the overlap between autism and EDs and, importantly, this lack of evidence base is leading to poor outcomes and inadequate support for Autistic people with an ED.

A promising avenue to address this is the use of co-production, which is a research approach that seeks to include those with lived/living experience as active collaborators in the research process. While there are several terms that can often be conflated or confused with co-production, such as participatory research, the underlying premise of these approaches is the inclusion of lived/living experience perspectives on what research gets done, how it gets done and how it gets shared [12, 31]. This can take many forms, including those with lived/living experience providing a consultatory role in research development and design, supporting recruitment and data collection, or helping to design and implement a dissemination plan for research findings. The most important principle of co-production however, is the importance of shared power and citizenship throughout the entire research process between those with lived/living experience and researchers (e.g., NIHR 42). It is this key principle of shared power dynamics that is central to conducting ethical co-production, whilst also being the principle that is frequently criticised as being undermined or unenforced (Rose and Katahil 54).

Co-production has been increasingly implemented in ED research, with researchers recognising the importance and value of lived/living experiences voices (Lewis and Foye 32). One of the leading drivers behind this is a lack of convincing evidence for ED treatments [56, 57], with the exclusion of lived/living experience perspectives in research proposed as a possible reason for these outcomes [49]. Lack of co-production has also been proposed to limit our understandings of other important ED considerations, such as ED illness and recovery trajectories [18, 19]. In the autism field, there have been similar observations on the lack of translation of autism research into meaningful change within the Autistic community [51], with the exclusion of Autistic perspectives proposed to be a notable cause for this [17]. There has been a shift amongst researchers highlighting the importance of consulting and collaborating with the Autistic community, engaging with and empowering Autistic individuals in the research process [15, 44, 59], and ensuring that autism research is conducted with or by Autistic people [40]. However, the field of EDs in Autistic individuals is in its infancy, and because of the two independent research fields working in relative isolation, there is a need to carefully consider the needs of this unique population. In theory, the benefits of co-production are multi-dimensional, promoting the inclusion of marginalised voices and seeking to empower those with lived/living experience to create and influence meaningful change. However, when it comes to actual application or implementation of co-production, there are several barriers that have been reported. In the ED literature, ethical co-production has been limited by a lack of clear definitions, leading to different or conflicting expectations [48], and a failure to adhere to the shared power dynamic [49]. Similarly, concerns regarding timelines, resources and a lack of clear guidelines on effective communication have been reported as possible barriers to adopting co-production [34, 52].

The Eating Disorders and Autism Collaborative (EDAC: [14]) is a UK Research and Innovation (UKRI)/Medical Research Foundation (MRF)/National Institute for Health and Care Research (NIHR) jointly funded project, that aims to enhance research capacity by supporting collaborations between the ED and autism research fields, with research activity being led by individuals with lived/living experience. To support this process and to build on previous literature on co-production in the independent ED and autism fields, the current paper seeks to provide insight into specific barriers to ethical co-production of research with Autistic people with an ED, and pragmatic considerations of how to navigate them. Therefore, the following study sought to address the following research question through online workshops and discussion: *What are the barriers and facilitators to ethical co-production with Autistic people with an ED?*

## Methods

### Collaborators

Study adverts were posted on EDAC’s social media accounts, as well as on the Scottish Women’s Autism Network (SWAN) and Scottish Autism online platforms. To be included in the study, participants (from here on referred to as collaborators), needed to identify as clinicians, researchers, parent/carers, autism or ED third sector representatives and/or as an Autistic person (self identified or formally diagnosed) with a diagnosed ED who was over 14, with the acknowledgment that many collaborators identified with multiple roles. In total, 30 collaborators took part in the workshops. Across collaborators 23.3% identified with multiple roles and 30.0% of Autistic collaborators with lived/living experience of an ED had received multiple ED diagnoses (see Table 1 for a summary of participant characteristics). Collaborators' names were not included to ensure anonymity and instead collaborators were assigned codes (e.g. EDAC1, EDAC2 etc.).

**Table 1:** Collaborator demographics (n=30).

Age (M, SD)	34.2 (9.92)	
Gender (%)	Female = 73.3%	
	Male = 13.3%	
	Trans/non-binary = 13.3%	
Ethnicity (%)	White = 90%	
	Mixed/multiple = 6.67%	
	Asian = 3.33%	
Location (%)	Scotland = 66.66%	
	England = 30%	
	Wales = 3.33%	
Roles (%)^a^	Autistic person with lived/living experience of ED = 66.67%	
	Researchers = 26.67%	
	Clinicians = 10%	
	Peer researchers = 6.67%	Multiple roles = 23.3%
	Third sector = 6.67%	
	Health/social care support role = 6.67%	
	Parent/carer = 3.33%	
EDs (N=20, %)^a^	AN = 70%	
	ARFID = 15%	
	BN = 15%	Multiple diagnoses = 30%
	BED = 5%	
	EDNOS/OSFED = 10%	

M = mean, SD = standard deviation

^a^Note that percentage totals in this column add up to more than 100% as many participants identified with multiples roles and had received multiple ED diagnoses

### Procedure

Ethical approval was obtained from the Research Ethics Committee at the University of Edinburgh (23-24CLPS183). Collaborators were asked to contact the research team if they wished to take part and eligibility was confirmed by email correspondence or through an online video chat. Once eligibility was confirmed, collaborators were asked to provide their consent and demographics. All collaborators provided informed consent to participate.

The overarching study involved five online workshops hosted over Microsoft Teams between December 2023 and February 2024 at two-week intervals. A Padlet (https://padlet.com) discussion board was shared each week to collect ‘offline’ thoughts and comments, and to offer an alternative method of engaging with the workshops. The Teams chat function was used during workshops to increase accessibility, and facilitators read out chat comments to the group to integrate into discussions. Recordings from the online workshops and comments from the Padlet discussion board were transcribed, integrated and anonymised, removing participant names and any other possible identifying information (names, locations, services, etc.).

The aim of the workshops was to better understand barriers and facilitators to co-production and to co-create best practice guidelines for ethical co-production for conducting research with Autistic people with an ED (see edacresearch.co.uk). The current paper focuses on the first two workshops, where barriers and facilitators to ethical co-production were discussed across the different stages of the research process.

### Data analysis

Transcripts were analysed using Reflexive Thematic Analysis (TA; Braun & Clarke, 7, Clarke & Braun, 2021) using an inductive approach. This approach was used to develop themes across the data, guided by our overarching research questions surrounding barriers and facilitators to ethical co-production. Transcripts were read and re-read, before early codes were identified. From these codes, themes were then developed and reviewed by a neurotypical and Autistic researcher with lived/living experience of an ED. This dual coding approach ensured that the generated themes accurately identified both the data and the lived/living experience of the many members of community involved in the study. The analysis approach was also aligned with the ethical co-production principles explored in the study, ensuring that Autistic people with an ED are involved as active members of the research team.

### Researcher reflexivity

It is important at this stage to reflect on the expertise and experience of the researchers involved in the analysis of the data. EN is a researcher who has conducted several research studies in the field of autism and EDs and has worked closely with the Autistic and ED community for several years. EM is an Autistic peer researcher with living experience of an ED and has been working across Autistic and ED community as well as research community for several years. Together, EN and EM co-produced the themes in the current study. It is worth noting that, as a research group, EDAC members were actively learning about co-production while running the workshops. At times in these workshops, the research team made mistakes and unwillingly fell into the traps associated with undermining co-production relationships. EDAC learnt from these mistakes and view the co-production process as an ongoing and reflexive process and acknowledge there is always more to learn.

## Results

### Barriers

**Fig 1:**
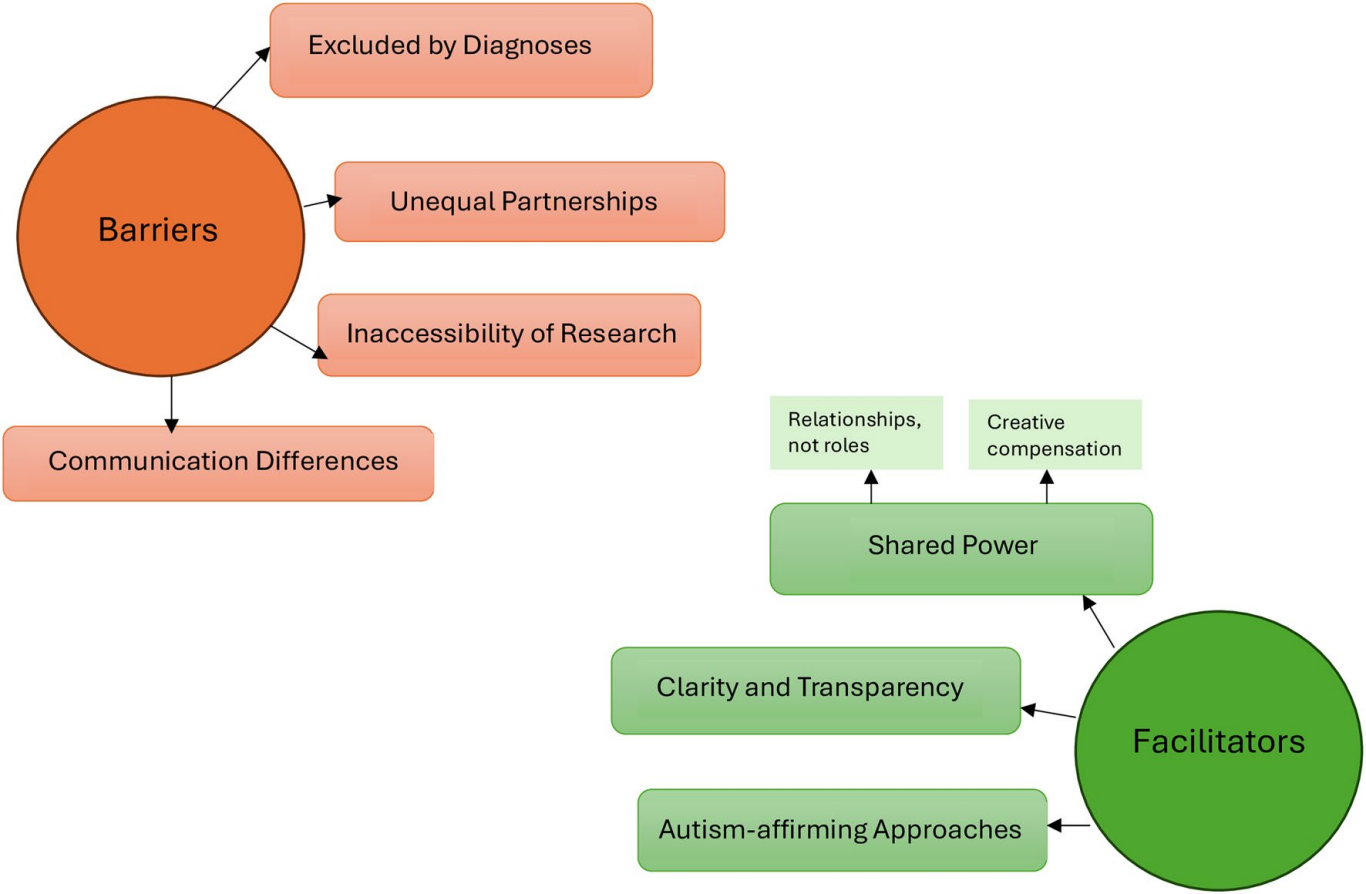
Thematic map of barriers and facilitators to ethical co-production

Thematic analysis generated four themes across the workshops regarding barriers to ethical co-production for Autistic people with an ED: *Unequal Partnerships, The Inaccessibility of Research, Excluded by Diagnoses* and *Communication Differences *(Fig. 1).

### Unequal partnerships

A leading theme discussed barriers within the dynamics of the co-production relationship. These discussions focused on inequality within the relationship between the lived experience role and the researchers, and the possible ways in which this can impact ethical co-production. Fundamentally, the leading barrier to co-production partnerships here is an imbalance of power. An example of how this balance of power can be skewed was through the involvement of lived experience after the research priorities had been set, or the studies had been designed:

Co-production [is] only sought after the direction of the research, predetermined by neurotypical researchers, and doesn’t align with meaningful priorities of Autistic individuals and communities.—EDAC8

Here, there is a lack of meaningful involvement, as true co-production involves a shared power, with individuals with lived experience actively involved from the start, and throughout, the research process. By involving lived experience perspectives at a later stage in the process, researchers run the risk of conducting research that is biased towards neurotypical-informed research objectives that lack the perspective of lived ED experience, as opposed to research that reflects the wishes and wellbeing of the autism and ED community. This unequal partnership was also discussed in the context of “*faux-production”*, or superficial way of engaging in co-production:

Just like faking it, bit like the tokenistic thing or tick box exercise….and it’s there no meaningful involvement.—EDAC9

In these instances, there is no partnership or shared power between the lived experience role and the researcher, as the lived experience perspective is not integrated into the research process meaningfully. This undermines the principles of co-production and makes those with lived experience feel undervalued.

A final consideration of this theme came from researchers themselves, who also discussed difficulties coming internally from members of the research team:

We have resistance from some researchers to share this decision-making power, which can be a huge barrier.—EDAC10

Resistance from researchers to share decision-making power adds an additional barrier to managing power dynamics within the partnership, where there can be internal inconsistencies or conflictions within research teams. In a similar vein, there are also infrastructural barriers to researchers that may impact their ability to conduct co-production to the best of their intentions:

One thing I’m a little concerned about is when we’re funded to do research, it’s not just the researcher’s ability or intention to make this happen, but its funders understand of that giving of power as well. And I think that’s quite, that can be quite difficult to navigate in these projects.—EDAC11

Thus, the barriers faced when managing power dynamics within the co-production partnership are multi-faceted, originating from different sources and pressures. This means that both those with lived experience and researchers must juggle navigating these barriers, whilst also ensuring that a collaborative and equal relationship is maintained.

### The inaccessibility of research

Another leading theme focused on the barriers that may be faced when engaging with or accessing research. Specifically, collaborators discussed several different ways in which they felt separated or isolated from research. Firstly, this was discussed in the context of being entirely unaware of what research is being conducted:

I don’t actually know much about the research that’s been conducted other than the summary that you guys have given. So, I don’t know how, you know, well informed I am to give an opinion on that, so it just feels a bit at odds with you know what you are asking—EDAC1

As many of those in the autism and ED community do not come from a research background, this highlights a foundational barrier to co-production, preventing those with lived/living experience from accessing, engaging and influencing research. This is a very important consideration, given that co-production is seeking to go beyond just *involving* lived/living experience in the research process towards *sharing* the research process and collaborating as mutual, active members of a research team. In a similar vein, a language barrier between researchers and the lived experience community was commonly discussed across collaborators This was discussed in different ways, such as the using of research jargon and acronyms without giving an explanation or definition:

You’re speaking two different languages as well. Not everybody with lived experience has experience of research, so you’re going to be speaking two very different languages and that’s a barrier...you’ve got to be speaking the same language in order to get something out of it—EDAC2

Not being able to understand or communicate with each other significantly impacts the co-production relationship. Overall, these discussions emphasised how much of a barrier this lack of shared language is, not only during the co-production process but, most importantly, in their possible engagement with it. For those who came to the process with more research experience, another barrier discussed was accessing it:

Research is really inaccessible, it’s behind paywalls, it’s in journals that are not accessible if you’re not part of a university or an academic institution—EDAC3

Access difficulties can mean that those in the autism and ED community are not able to read or share any research, or gain information about who is conducting the research. This is a clear barrier to engagement with co-production and reinforces a sense of separation between researchers and those in the lived experience community. For one participant, being unable to find or access research on the overlap between autism and ED made her doubt her own experiences:

I was looking to try and have kind of some evidence to try and get, you know, the process going, and I really struggled to find anything…I started like almost doubting it. I was like, maybe I’m wrong, maybe there isn’t actually a link between eating disorders and autism—EDAC4

Therefore, if the research is not made accessible to the autism and ED community, individuals will be unaware what research is being done, what research they would like to get involved with and even if they are eligible to engage with the research.

### Excluded by diagnoses

A third theme that was discussed by many collaborators was the limitations that current autism and ED diagnostic criteria can have on ethical co-production. This was highlighted by one participant, who stated:

I’ve not felt either Autistic enough or that I’ve had a bad enough eating disorder…I wouldn’t necessarily put myself forward—EDAC3

In some cases, it could be that studies that fail to advertise clearly and specify what the essential inclusion criteria are that gives rise to confusion. However, across collaborators, this theme was particularly felt to be specific to their experiences as an Autistic person with an ED. This could come from feeling like their autistic characteristics were not “significant” enough, but particularly came from discussing how current ED diagnostic criteria do not map onto their experiences:

People are being included in studies without many having discussions about why people behave in ways they do, or maybe just measured against diagnostic criteria rather than lived experience, which can be quite different—EDAC2

Thus, collaborators felt that current diagnostic measures do not align with autistic experiences of EDs, which leads Autistic individuals to feel excluded or unable to take part in co-production. Some collaborators discussed why this may be the case, reflecting on how current diagnostic approaches are based on neurotypical presentations of EDs, similarly emphasising the importance of understanding different experiences:

The labels in the terms we use in our understanding of eating disorders is very much from a neurotypical lens...it’s like we are putting these frameworks on Autistic people rather than thinking about like, these behaviours look quite similar in Autistic people but the actual experience but might be entirely different.—EDAC5

Therefore, relying on strict diagnostic criteria, or stereotyped assumptions of EDs or autistic presentations, could potentially lead to restrictive research explorations and excludes an array of experience. It is imperative that this nuance and variance is captured, leading to research outputs that represent and resonate with the wider community.

### Communication differences

Finally, collaborators also commonly reflected on the impact that communication differences can be a barrier to engaging with co-production. This was mainly felt to manifest through a lack of understanding of, and adjustment for, autism-specific communication differences. This could be down to different ways or methods of communication, such as those who are non-speaking commonly being excluded from co-production roles due to their focus on the spoken word:

A lot of work is, you know, requires a level of literacy and or digital inclusion and that does exclude people who might be supported in social care or have a learning disability as well.—EDAC6

Another key point raised here was that of ‘digital literacy’, and of the potential bias in co-production roles towards those who are comfortable with, or have access to, online technology and social media:

If you’re not actively online in these spaces, it’s going to make it hard to [do] co-production with a diverse range of people and ethically do it too—EDAC3

This raises an important barrier to ethical co-production through its possible exclusion of a significant range of Autistic experiences, leading to a lack of diversity in co-production positions. Additionally, not only were communication barriers discussed with regards to accessibility and the need to consider different methods/platforms of communication, but many collaborators discussed the impact of these differences. Specifically, many Autistic individuals felt that the content of their communication was poorly understood by non-Autistic people:

I’ve had difficulties with being understood or misinterpreted by others before. For example, people said I’ve been rude or inappropriate when I’ve been thinking I was being direct and I wonder if others maybe struggle to want to put themselves forward for these things out of feeling they [would be misunderstood]—EDAC3

This lack of awareness and understanding of communication differences can be a barrier to the co-production partnership in many ways. Here, the collaborators discussed it within the context of engaging with co-production roles, suggesting that these misunderstandings are common and harmful enough to possibly lead to a lack of trust, and many Autistic individuals not wanting to put themselves forwards in the first place. This was unfortunately a common experience across collaborators, with one participant reflecting that this led to her feeling “*dismissed*” and “*undervalued”,* and that this could be a barrier to engaging in co-production as “*it was quite a traumatic experience and so people might be reluctant to come forward and sort of share afterwards and that, you know, feel quite anxious about that.” EDAC7*

Collaborators also discussed the importance of not only being aware of these barriers but of how these barriers may look different across different individuals:

It’s important to recognise the barriers that Autistic people may face in expressing their views and accommodating, you know, their participant in a range of different ways. So, not thinking, you know, one approach is going to be appropriate for everybody—EDAC1

Fundamentally, failing to understand and adapt for the range of communication preferences and styles will pose a significant barrier to ethical co-production, excluding a significant number of Autistic individuals and experiences from research.

### Facilitators

Three themes were identified surrounding Facilitators of ethical co-production. These were *Shared Power (*with two sub-themes of *Relationships, Not Roles* and *Creative Compensation), Clear Communication, Autism-Affirming Approaches.*

### Shared power

Mirroring the final theme regarding barriers to ethical co-production, a leading theme felt to facilitate ethical co-production was creating empowered partnerships between those with lived experience and the researchers. One sub-theme here was *Relationships, Not Roles,* which focused on the nature of the partnership itself. There were several different ways that collaborators felt that power could be shared within the partnerships; for example, involving Autistic people with an ED from the very beginning of the project, and putting time into developing relationships, as opposed to just using lived experience to fill roles:

Need to get people [Autistic people with EDs] involved in the research from the get-go, where it is important to establish relationships—EDAC10

Equally as important was maintaining this involvement across all stages of the research process, with *“true collaboration [being] from start, middle to finish*” *(EDAC9)*. The same participant went on to emphasise the importance of being led by experience and having Autistic people with an ED in positions of leadership:

It’s really helpful having those lived experiences embedded as kind of part of the leader of, like leading and facilitating groups. So about holding that power…I think that can really help and empower lived experience and it shows a good example of good practice to other organisations—EDAC9

Having Autistic people with an ED in more primary, leading roles within the partnership, allows for this shared and balanced sense of power that is integral to a successful and ethical co-production relationship. This was another important dimension to empowering the partnership; the emphasis on the relationship and the importance of maintaining that over time. Collaborators discussed that these partnerships should be flexible and adaptive, as well as emphasising that they are bi-directional:

There is something about evaluation and feedback as well, so that there needs to be feedback loops going both ways…and maybe relevant review points as well to check how that relationship is feeling for both sides and with an honesty and acknowledgement that if it’s not right, we don’t just carry on, we do something [about it]—EDAC1

To facilitate ethical co-production, it was felt important to view the process as an ongoing and dynamic relationship and not merely as a role, helping to empower those with lived experience and establish shared power in the partnership.

Another important aspect of shared power that was discussed in a second subtheme was making sure that those with lived experience are reimbursed for their time *(Creative Compensation)*. Collaborators discussed instances where they had engaged in research and felt undervalued, while researchers discussed difficulties obtaining money from funders to financially compensate these roles. While the consensus was that financial compensation should be offered where possible – and effort should certainly be made to obtain this – the group talked about different ways where those with lived experience can be reimbursed for their time:

A lot of us are passionate and want to be involved, money is great but it isn’t everything. Having the results of the study, being asked to be in research more or being an active participant or research team member. Groups which continue long term (even with no payment) can create connections, networks and support—EDAC3

Again, there is the keen sense that developing empowered partnerships and relationships are a leading facilitator in ethical co-production. Ensuring that these relationships are maintained over time and continued support is offered can, in some instances, be a good interim approach to making sure those with lived experience feel valued in their input. One participant raised the point that a lack of funding should not stop researchers from engaging with co-production:

Just kind of getting a bit creative with what opportunities there are, because I think that a lack of funding for research shouldn’t be a barrier to co-production—EDAC5

Therefore, for some individuals, there may be scope to discuss alternative methods of compensating them for their input. However, this may be down to personal circumstances and there may be certain individuals in more of a position to be flexible with this compensation. Fundamentally, financial compensation and ensuring that those with lived/living experience are paid and valued for their time should be the primary strategy, serving to reinforce their role of an active member of the research team.

### Clarity and transparency

A second theme that was identified across the workshops was the need for clear and consistent communication to successfully facilitate ethical co-production. For many, this began with establishing clearly defined roles within the research team:

There’s something really important whenever we are talking about co-production about setting clear expectations and parameters for both sides. And I think in doing that, it allows accessibility—EDAC1

By making sure that all parties clearly understand the requirements and expectations from the role, those with lived experience may feel more comfortable engaging in the co-production process. It will also improve the accessibility of the research and work towards including a more diverse and representative range of individuals in co-production roles. The importance of clear communication was also discussed in the context of communicating about the research itself:

The purpose and the audience for any research…who’s the research aimed at, what purpose is it hitting? What outcomes do we expect?—EDAC1

It was felt to be important that, when co-producing research, all members of the research team clearly understand the rationale behind the research and what to expect from the results of the research. Importantly, many collaborators also focused on how translatable the research would be, and how important it is to clearly understand the implications, and communicate how the results would directly impact the lives of Autistic people with an ED; this was consistently felt to be a leading research priority.

In a similar vein, collaborators also discussed the importance of communication styles:

Just the word ‘clear’, about making sure everything is really clear and explicit, read over by experts by experience to help with that maybe, so there’s nothing ambiguous as far as possible or that may be misinterpreted—EDAC3

This latter quote highlights an important point surrounding possible differences in communication between Autistic and non-Autistic people, and the need for Autistic people to be involved in developing or reviewing communications to remove the risk of misinterpretation between the two neurotypes. Collaborators also discussed the importance of sharing this information in multiple different formats, accounting for the heterogenous needs of Autistic communication. Examples included sharing information in audio or verbal formats, with plain English, alternative text and read-out-loud options, as well creating videos or sharing pictures or visualisations.

### Autism-affirming approaches

The final theme was the need to adopt autism-affirming approaches when conducting ethical co-production. This was discussed across several different contexts, with collaborators emphasising the importance of moving away from the harmful medical and deficits-based approaches of the past, towards an approach that listens to and is led by the Autistic community, seeking to conduct research that is meaningful and directly translatable to improve the lives of Autistic individuals with an ED. For example, previous research was felt to use “*dehumanizing language*”, or have sought to “*fix Autistic people”.* Instead, one participant said that:

Do research that Autistic people actually want, not what parents want or like medical professionals want about, like, getting rid of autism or Autistic people, but like, how do you improve the lives of Autistic people?—EDAC5

Thus, ethical co-production should involve Autistic people with an ED in guiding the direction of research, making sure research aims and findings reflect the wishes of the autism and ED community, and are focused on directly improving support and care for this community. This ensures a shift away from deficit-based approaches towards a strengths-based approach, in both research priorities and in research culture.

Another example by which autism-affirming approaches should be used to facilitate ethical co-production is by focusing on the wellbeing of collaborators and of those with lived experience involved in the research process. This was felt to be an important consideration when drawing on lived experience perspectives in co-production particularly:

I think it’s about the wellbeing…so if you’re just delivering and sharing your experiences constantly without backup from the right support, [it] can be quite overwhelming. So, it’s just of making sure that there’s systems before, during and after—EDAC12

While these roles are designed for those with lived experience, the expectation should not be that those who come forward for these roles can discuss such experiences without impact. Everyone’s ED journey is different and reflecting on this is likely to be distressing at points for the individual. This participant highlights that the correct support should be put in place before the co-production process begins, during the process and after the role has finished. This feeds back into the idea that ethical co-production involves meaningful involvement and relationships across the entire research process. By acknowledging and accounting for this possibility, researchers can ensure that those with lived experience feel safe and supported in their role, and can truly work to facilitate ethical co-production.

## Discussion

The current study sought to explore possible barriers and facilitators to ethical co-production with Autistic people with an ED, in a group reflecting research, clinical, community and lived/living experiences. Leading barriers were difficulties with access, current ED and autism diagnostic criteria, and communication, underpinned by the reinforcement of unequal power dynamics and partnerships. Mirroring this, key facilitators were sharing power in the co-production relationship and clear communication, operating within an autism-affirming approach. Key findings will be discussed below, and the implications of study findings will be discussed in the context of how to ensure future co-production is conducted ethically and is translated to improving the lives of Autistic people with an ED.

One of the most important, and arguably undermining, barriers to ethical co-production was unequal power dynamics. Study findings suggest that co-production can only be considered meaningful, and thereby ethical, to those involved if researchers engage in shared power with those with lived/living experience. This has been found to be a universal facet of co-production [12, 52, 54], however it is important here to consider the unique implications within the field of autism and ED. Historically, Autistic voices have been excluded from autism research [17], leading to research being conducted that has been, at best, misaligned with community priorities and, at worst, causing harm to the Autistic community. A similar absence of lived/living experience voices has been reported in the ED field [49]. Mirroring this, one of the leading facilitator themes in the current study suggested that, to conduct true ethical co-production, researchers need to address this history of mistrust and exclusion, creating equal partnerships of shared power and decision making. These findings are in line with emerging guidelines for co-production in autism [44, 59] and ED [49] research, and should be at the heart of all future co-produced research with Autistic people with an ED.

At the entry level of co-production, the current study found that Autistic people with an ED felt current research was inaccessible on multiple levels. Many felt isolated from research due to an inability to access papers through university or academic paywalls. This was interesting, as it presented a barrier at the very conception of our discussions, raising the question of how to engage in discussions regarding barriers and facilitators to ethical co-production if one is blocked from accessing the research that has been conducted. The publication of research in open access journals and platforms is being promoted across contexts, allowing for rapid and more widespread access of findings [62]. Furthermore, researchers should consider sharing their findings in alternative ways, including community outreach, such as knowledge exchange (KE) events, or making research accessible to the community it serves via different mediums e.g. infographics, podcasts, etc. Additionally, the inaccessibility of language was also discussed in the context of speaking two different ‘languages’ and the need to work towards a shared research language. In the current context of autism and ED research, an important consideration here will be the use of key terms. While language preferences may change across individuals, there is the consensus amongst ED literature to avoid language that implies the person is defined by their ED, such as referring to a person as an ‘anorexic’ or ‘bulimic’ [64]. The autism literature posits the opposite, shifting away from the medicalized model of autism that emphasise ‘deficits’ and the need to ‘cure’, towards identity-first (Autistic, rather than person with autism) and difference-based language [5, 10, 23]. This was also central to the facilitator theme of autism-affirming approaches, where it was felt that this shift would lead to research that reflects the actual priorities of Autistic people with an ED and that seeks to improve, not eradicate, the lives of Autistic people. Researchers engaging in co-production should thus be aware of these discussions and engage with Autistic people with an ED to develop this shared and humanizing language.

Another leading barrier discussed was the limitations of diagnostic criteria that are often enforced within research. This was particularly relevant in the context of ED diagnoses, which were felt to reflect neurotypical experiences and not Autistic experiences of EDs. It remains a field-wide issue that the underlying mechanisms of EDs in Autistic people remain poorly understood. It could be that difficulties experienced with current diagnostic criteria reported here, may partially explain this lack of understanding, with researchers and clinicians approaching these attempts to understand from a neurotypical perspective. For instance, researchers could be using ED diagnoses as an inclusion criterion in research or use ED measures that have been developed in neurotypical populations [20, 35, 61]. In the current context, this was felt to lead to Autistic people feeling excluded from, or unable to take part in, co-production. Thus, while this may not always be possible (for example, in controlled study designs that require stringent criteria), to ensure that researchers are including true Autistic experiences in co-production, the scientific community will need to adopt a broader and more reflexive understanding of EDs in Autistic people, rooted in lived/living experiences. This again reflects one of the leading facilitator themes highlighted in the study, the importance of operating under an autism-affirming approach and working towards conducting research that reflects and supports the priorities and wellbeing of the Autistic and ED community.

Another key element to ethical co-production that was discussed as both a barrier and a facilitator to co-production was that of communication. Barriers were included information about research and co-production opportunities being shared in limited ways (e.g., depending on a level of literacy) and on limited platforms (e.g., depending on access to online spaces). Communication differences were also discussed with regards to neurotypical researchers misunderstanding or misinterpreting Autistic communication, in line with recent perspectives that reframe traditional social communication ‘deficits’ in autism into bi-directional miscommunication between Autistic and neurotypical individuals [13, 39]. In line with this, a leading facilitator of ethical co-production was clear, transparent and accessible communication, facilitated by the involvement of an Autistic person with an ED as an active member of the research team. This two-sided nature of communication differences is a core element of previous studies that have explored experiences of co-production in autism (e.g., [52, 59]), and should be strongly considered when approaching co-produced autism and ED research. This will require researchers to understand and adjust for communication differences, and to carefully develop recruitment strategies that seek to reach the full and diverse range of Autistic experience. This will also be integral to ethical co-production within the research team, ensuring effective communication within teams where those with lived/living experiences are active members or in leadership roles.

The current study has several notable strengths. Firstly, workshops were run with a broad range of experiences and perspectives. This included researchers, peer researchers, clinicians, those that work in the third sector, parents and/or carers, and Autistic individuals with lived/living experience, with around 23% of individuals self-identifying as having multiple roles. By working with a diverse group of individuals, we sought to collect the experiences of all key stakeholders in the autism and ED field, with a focus on roles that would typically be involved in the co-production process. Another strength of the paper was that the themes were co-produced with an Autistic peer researcher with lived/living experience of an ED, as was the entire study, from conception to write up and submission. It is also noted that Autistic collaborators consisted of both individuals who self-identified and those with a formal autism diagnosis. This was an active, and co-produced methodological decision, taking into account the barriers and long waiting lists to access diagnostic assessments. The study also recruited a range of ED diagnoses and experiences, although it should be noted that there was a notable bias towards anorexia nervosa (70%) in the study group, which is echoed in the broader autism and ED literature (e.g., [16, 36]). While current findings highlighted concerns surrounding the use of these diagnostic groups, future research should seek to recruit a more balanced range of ED experiences, shifting away from a restrictive eating focus. Similar limitations can also be observed with regards to gender and ethnicity, with a stark bias towards white (90%) and female (73.3%) collaborators. This gender bias has been frequently cited amongst ED literature [9], and the increasing shift towards recognising and understanding EDs in men [41, 60] and transgender and non-binary individuals [38] should be adopted in future autism and ED co-production.

It is hoped that current findings will shed novel insights and inspire creative and respectful collaboration in future co-produced research projects in the field of autism and EDs. By highlighting several areas that may pose barriers to ethical co-production or should be focused on to facilitate ethical co-production, these findings and reflections will hopefully form a foundation for co-production research teams to engage in discussions in how to best approach, conduct and share co-produced research. Through such approaches, it is hoped that future research will address current limitations of the literature, moving towards evidence-based and co-produced understandings of the overlap between autism and EDs; what may be underlying this overlap and, most importantly, translating these understandings into improving support and wellbeing of Autistic people with lived/living experience of an ED. While we have sought to provide suggestions on how to address barriers and facilitators to ethical co-production, it should be noted that these should be used as a template or foundation for future projects, as opposed to instructions. The specific context and dynamics of research projects will vary dramatically, and the key message here is to engage meaningfully with those with lived/living experience and enter the process as an equal partnership. As part of the wider project, researchers and collaborators at EDAC co-produced a set of Best Practice Guidelines for Ethical Co-Production, which goes through the different stages of the research process as well as providing examples from brain imaging and arts-based methodologies (see edacresearch.co.uk)

## Conclusions

Historically, research has been done to, not with, Autistic people with an ED. Co-production represents a fundamental shift in this dynamic, but only if this is done ethically. To do this, co-production teams will have to collaboratively navigate a myriad of potential barriers, such as imbalanced power dynamics, communication differences and applying neurotypical ED criteria to Autistic ED experiences. Current findings suggest that key to this successful facilitation of ethical co-production will be creating shared and equal longer-term partnerships, communicating with clarity and transparency within an autism-affirming framework. It is hoped that current findings will be used to encourage novel and truly collaborative research with the Autistic and ED community, driven by the priorities and experiences of Autistic people with lived/living experience of an ED.

## Data Availability

The datasets used and/or analysed during the current study are available from the corresponding author on reasonable request and subject to ethical approval.

## Abbreviations

AN: Anorexia nervosa
ARFID: Avoidant restrictive food intake disorder
BED: Binge eating disorder
BN: Bulimia nervosa
ED: Eating disorder
EDAC: Eating disorder and autism collaborative
EDNOS: Eating disorder not otherwise specified
OSFED: Other specific feeding and eating disorders
